# Inhibitory Effects of Citrus-Derived Flavonoids Hesperidin and Hesperetin on SARS-CoV-2 Spike-Mediated Syncytia Formation Using In Vitro Cell Model

**DOI:** 10.34172/apb.44060

**Published:** 2025-03-09

**Authors:** Dennaya Kumara, Hayfa Salsabila Harsan, Endah Puji Septisetyani, Pekik Wiji Prasetyaningrum, Komang Alit Paramitasari, Mukh Syaifudin, Okid Parama Astirin, Muthi Ikawati, Edy Meiyanto

**Affiliations:** ^1^Cancer Chemoprevention Research Center, Faculty of Pharmacy, Universitas Gadjah Mada, Yogyakarta, 55281, Indonesia; ^2^Mammalian Cell Engineering Research Group, Research Center for Genetic Engineering, National Research and Innovation Agency (BRIN), West Java, 16911, Indonesia; ^3^Research Center for Radioisotope, Radiopharmaceutical and Biodosimetry Technology, Research Organization for Nuclear Energy, National Research and Innovation Agency (BRIN), Banten 15310, Indonesia; ^4^Department of Biology, Faculty of Mathematics and Natural Science, Universitas Sebelas Maret, Surakarta 57126, Indonesia; ^5^Laboratory of Macromolecular Engineering, Department of Pharmaceutical Chemistry, Faculty of Pharmacy, Universitas Gadjah Mada, Yogyakarta, 55281, Indonesia

**Keywords:** COVID-19, Citrus, Flavonoids, SARS-CoV-2, Syncytia

## Abstract

**Purpose:**

SARS-CoV-2 infection may lead to a worse prognosis in COVID-19 patients by inducing syncytia formation which implies intercellular transmission and immune evasion. Hesperidin (HSD) and hesperetin (HST) are two citrus flavonoids that demonstrate the potential to interfere with spike/human angiotensin-converting enzyme-2 (hACE2) binding and show an inhibitory effect in the SARS-CoV-2 pseudovirus internalization model. Here, we determined the effects of HSD and HST to inhibit syncytia formation using in vitro cell models.

**Methods:**

We confirmed spike, hACE2, and transmembrane protease, serine 2 (TMPRSS2) ectopic expressions by immunofluorescence staining (IF) after transfection using polyethylene imine (PEI) in 293T cells. Then, the cells were transfected with a set of plasmids encoding spike/hACE2/TMPRSS2 or spike/hACE2 to induce syncytia formation. Cell treatment with HSD/HST was performed 4-5 h after transfection and then incubated for another 16-18 h. Syncytia were observed using an inverted microscope or a high content screening (HCS) platform. The data obtained from syncytia formation assays were statistically analyzed using ANOVA (Bonferroni).

**Results:**

We successfully observed spike, hACE2, and TMPRSS2 expression in 293T cells by IF staining. Furthermore, we showed that HSD 10 and 100 µM significantly inhibited the formation of small-to-medium-sized syncytia compared to the control cells by manual syncytia observation. In the HCS assay, 10 µM HSD showed an inhibitory effect of syncytia induced by spike WT. In contrast, 100 µM HSD, 10 and 100 µM HST, and 10 µg/mL citrus peel extract containing HSD prepared by the hydrodynamic cavitation method (HCV) inhibited syncytia formation induced by spike Omicron.

**Conclusion:**

HSD and HST show the potential inhibitory activity of SARS-CoV-2 intercellular transmission. Further study is needed to confirm the mechanism of action of the antiviral activity.

## Introduction

 The coronavirus disease 2019 (COVID-19) has recently caused public health concerns. Ignited by severe acute respiratory syndrome coronavirus 2 (SARS-CoV-2) infection through human angiotensin-converting enzyme-2 receptor (hACE2) binding, COVID-19 generates symptoms of human respiratory cell infection.^1,2^ While viral entry mechanisms have been reported to be mediated by membrane fusion and endocytosis,^3^ further infections may occur through intercellular transmissions by forming multinucleated cells known as syncytia.^4^

 Intercellular transmissions are made possible when spike protein on the surface of infected cells interacts with hACE2 on the neighboring cells, mediated by the presence of transmembrane protease, serine 2 (TMPRSS2).^5,6^ These interactions cause fusions of infected cells with other host cells, leading to syncytia formation.^6^ Syncytia were proved to be found in lung tissues of early-stage COVID-19 patients.^7^ Bussani et al^8^ in their systematic analysis of COVID-19 patients report that post-mortem examination showed the presence of syncytia in 36 out of 41 patients. Syncytia are susceptible to apoptosis and pyroptosis, enabling the release of the virus, which leads to viral dissemination and inflammatory response activation.^6^ More importantly, syncytia enable the virus to spread directly from cell to cell, thereby protecting it from immune cells and physical barriers.^6^ Therefore, the implications of syncytia on SARS-CoV-2 infections reinforce the importance of a therapeutic candidate that can inhibit syncytia formation.

 Hesperidin (HSD), an abundant flavonoid glycoside found in citrus peel,^9^ has potential antiviral properties against SARS-CoV-2. Among the major constituents of natural resources, we previously found that HSD has the strongest interaction with proteins related to SARS-CoV-2 infection in silico.^10,11^ Additionally, as reviewed by Agrawal et al.^12^ HSD has shown in vivo antiviral activity toward several viruses such as encephalomyocarditis virus, rotavirus, and herpes simplex virus-2. HSD exhibits a higher binding affinity to proteins, specifically spike, ACE2, and TMPRSS2, than the previously used antivirals in COVID-19 therapy, lopinavir, nafamostat, and camostat.^10,11^ Hesperetin (HST), the HSD aglycon, is also present in the citrus peel, but at a lower amount than HSD. HST may be formed via HSD enzymatic glycolysis in our intestine. HST also has been studied for its potential as an anti-SARS-CoV-2. HST can interfere with the interaction of the ACE2 receptor and TMPRSS2 by strongly interacting with ACE2, even more so than chloroquine, thereby hindering ACE2’s interaction with the receptor-binding domain (RBD) of the SARS-CoV-2 spike glycoprotein.^10^ Specifically, docking simulations show that HST strongly bind to the ACE2 receptor.^12^ In-silico investigations provide evidence of HST’s inhibition of the hACE2-spike glycoprotein complex of SARS-CoV-2.^13^ Since SARS-CoV-2 spike, hACE2, and TMPRSS2 play important roles in syncytia formation, thus, HSD and HST are expected to show antiviral effects through the inhibition of syncytia formation.

 This study investigates the ability of HSD and HST to inhibit syncytia formation. To perform the syncytia assay, we used 293T cells transfected with plasmids for spike/hACE2/TMPRSS2 co-expression. We also observed the effect of both flavonoids and HSD-containing extract obtained by HCV on the syncytia formation mediated by the SARS-CoV-2 wild type (WT) and Omicron spikes by utilizing the high content screening (HCS) instrument with the lifeact-GFP as a biosensor. The number of syncytia following each treatment, compared to the DMSO controls, demonstrated the inhibitory potential of HSD and HST.

## Materials and Methods

###  Cell culture

 The 293T cells (ECACC 12022001) and BHK-21/WI-2 (Kerafast EH1011, USA) were collection of the National Research and Innovation Agency (BRIN, Indonesia). The 293T cells were grown in high-glucose Dulbecco’s modified eagle’s medium (DMEM) (Sigma Aldrich, St. Louis, USA) supplemented with 10% fetal bovine serum (FBS) (Biosera, Cholet, France) and 100 IU/mL penicillin/100 mg/mL streptomycin (Gibco, Billings, USA), while BHK-21 cells were grown in 10% FBS/DMEM medium. Cells were incubated at 37 °C in a humid incubator with 5% CO_2_.

###  Tested materials

 HSD (Sigma PHR1794) and HST (Sigma SHBL8821) were dissolved in 100% dimethyl sulfoxide (DMSO) to prepare a 50 mM stock solution stored in aliquots at -20 °C. In addition, citrus (*Citrus reticulata*) peel extract prepared by the hydrodynamic cavitation method (HCV)^14^ was also dissolved in DMSO to obtain a 50 mg/mL stock solution. The concentration series of tested materials in a culture medium with DMSO as a co-solvent was freshly prepared before cell treatment. The final solution of 10 and 100 μM HSD/HST contained 0.1 and 1% DMSO, respectively, thus we used DMSO 0.1 and 1% as co-solvent controls for each treatment.

###  Recombinant plasmids

 Plasmid for ectopic expression of SARS-CoV-2 spike glycoprotein (pcDNA3.1-SARS2-Spike; Addgene #145032; a gift from Fang Li),^15^ hACE2 (pCDNA3.1-hACE2; Addgene #145033; gift from Fang Li),^15^ or TMPRSS2 (TMPRSS2 plasmid; Addgene #53887; a gift from Roger Reeves),^16^ were obtained from Addgene (US) as bacterial stabs (spike and hACE2) or purified plasmid (TMPRSS2). Lifeact-green fluorescent protein (GFP) as an actin cytoskeleton biosensor (pEGFP-C1 Lifeact-EGFP; Addgene #58470; a gift from Dyche Mullins),^17^ was obtained from Addgene as bacterial stab.

###  Plasmid maxi-prep

 TMPRSS2 plasmid was introduced into *Escherichia coli* (*E. coli*) DH5α competent cells prepared by CaCl_2_. Recombinant *E. coli* was stored as a frozen stock in LB/30% glycerol at -80 °C in a deep freezer. In addition, the bacterial stabs obtained from Addgene were streaked on an LB/Amp plate to obtain single colonies, which were further picked up, re-grown as suspension culture, and stored as frozen stocks. The bacteria’s frozen stocks were used for cloning from small-scale culture (~3 mL) to 400 mL scale culture in LB medium containing 50 mg/L ampicillin in an incubator at 37 °C with shaking at 150–180 rpm. The plasmid was purified from the cell pellet using a plasmid maxiprep kit (Qiagen), then dissolved in TE buffer, and its concentration was measured using a microvolume spectrophotometer (Nanodrop, Thermo Fisher Scientific) to determine the concentration.

###  Cell transfection and immunofluorescence (IF) staining

 The 293T cells were grown on gelatin-coated cover glasses at 30 000 cells/well density in a 24-well plate. The following day, cells on different wells were separately transfected with 1 µg of pcDNA3.1-SARS2-Spike,^15^ pCDNA3.1-hACE2,^15^ or TMPRSS2 plasmid,^16^ by using 3 μL of 1 mg/mL polyethyleneimine (PEI-Max, Polysciences) transfection agent.^18^ The next day, cells were fixed with 4% paraformaldehyde (PFA) for ~20 minutes, permeabilized with 0.2% Triton X-100 for 10 minutes, and incubated with a blocking buffer (1% bovine serum albumin (BSA)/phosphate buffered saline (PBS)) for approximately an hour. Cells on the coverslip were then incubated with anti-spike glycoprotein antibody (Abcam ab275759, rabbit polyclonal), anti-hACE2 antibody (Sigma SAB3500977, rabbit polyclonal), or anti-TMPRSS2 antibody (Bioss bs-6285R, rabbit polyclonal) by the hanging drop method overnight at 4 °C. After probing with primary antibody, secondary antibodies conjugated with a fluorochrome were added and incubated for a minimum of 3 hours to visualize the antigen-antibody reaction (Abcam ab150077 Alexa Fluor®-488 conjugated anti-rabbit antibody or Alexa Fluor®-594 conjugated anti-rabbit antibody). The DAPI-containing mounting medium was dripped onto the object glass to mount the cells on the coverslip. Protein expression was investigated using a motorized fluorescence microscope (Olympus IX83, Japan).

###  Syncytia formation assay

 The 293T cells were seeded at a density of 70,000 cells/well in a 24-well plate and incubated overnight (37 °C, 5% CO_2_). Cells were transfected with 0.5 µg pcDNA3.1-SARS2-Spike, 0.25 µg pcDNA3.1-hACE2, and 0.25 µg TMPRSS2 plasmid by using 3 µL of 1 mg/mL PEI. Cells were incubated for about 6 h (37 °C, 5% CO_2_), and the transfection medium was replaced with HSD or HST 10 and 100 µM in two replicates. After 24 h post-transfection, cells were washed three times with PBS and then fixed with 300 µL of PFA for ~20 minutes. Syncytia were observed under an inverted microscope, and ten microscope fields were taken for each replicate for manual analysis of syncytia.^19^

###  Syncytia assay using HCS platform

 A syncytia assay was performed using a co-transfection system for a single cell population.^20^ The 293T cells were seeded in an optical-bottom black 96-well plate (Nunc, Thermo Fisher Scientific, USA) at a 1.4.10^5^ cells/mL density. The next day, co-transfection of SARS-CoV-2 spike, hACE2, and lifeact-GFP encoding plasmids was performed for about 6 hours. Then, the medium was replaced with the test solution in triplicate and incubated for about 16–18 hours. The cells were stained with Hoechst for nuclei staining and then observed using a Cellinsight CX7 LZR high-content screening instrument (Thermo Fisher Scientific, USA). Nine fields per well were acquired to analyze syncytia numbers based on HCS analysis for the colony formation assay. For channel 1, the Hoechst indicator was selected for nuclei analysis, and for channel 2, the GFP indicator was selected to investigate GFP-positive cells with a GFP threshold determined automatically. GFP-positive cells with 2 or more nuclei were categorized as syncytia.

###  Statistical analysis

 The significance of the reduction of syncytia formation was analyzed using one-way ANOVA with Bonferroni correction. *P* values < 0.05 were considered statistically significant.

## Results and Discussion

###  Expression of SARS-CoV-2 spike, hACE2, and TMPRSS2 

**Figure 1:**
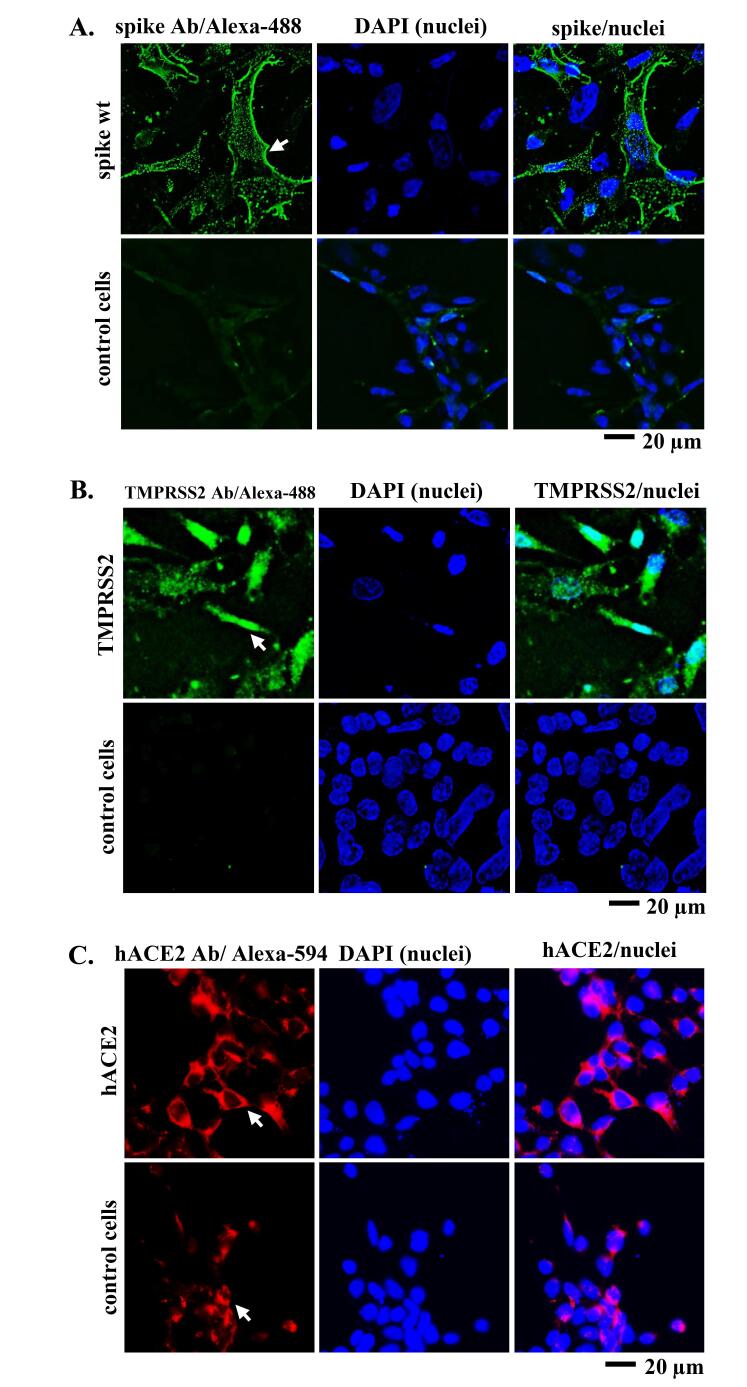
IF staining of 293T cells that were previously transfected with spike, hACE2, and TMPRSS2 plasmids. (A) Spike (green), (B) TMPRSS2 (green), and (C) hACE2 (red) expression were observed at 24 h post-transfection. White arrow denotes Alexa-488 and Alexa-594 signals representing spike, hACE2, or TMPRSS2 expression as indicated

 Apart from direct viral entry from the outer milieu into the target cell, viral entry can also occur through secondary infection by intercellular transmission. In the case of the SARS-CoV-2 virus, this can occur due to the interaction of the spike expressed by the infected cells with hACE2 of the neighboring cells in the presence of TMPRSS2, which gradually promotes the fusion of the two cells. Therefore, we performed IF staining to confirm the expression of spike, hACE2, and TMPRSS2 following plasmid transfection. Anti-spike and anti-TMPRSS2 primary antibodies bind to the secondary antibody conjugated with Alexa-488. Meanwhile, anti-hACE2 primary antibodies bind to the secondary antibody conjugated with Alexa-594. It enables the detection of green signals originating from the Alexa-488 and red signals originating from the Alexa-594 fluorophore. The mounting medium contained DAPI, to stain 293T cell nuclei blue. Green, red, and blue fluorescence signals were observed using a fluorescence microscope. Cell microscopy revealed green and red fluorescence signals surrounding the nucleus (Figure 1), indicating the successful expression of spike, hACE2, and TMPRSS2 in 293T cells. Compared to the control cells, the green and red signals in the transfected cells were stronger and more evenly distributed and localized in the cytoplasm or cell membrane.

###  SARS-CoV-2 spike-induced syncytia formation

**Figure 2:**
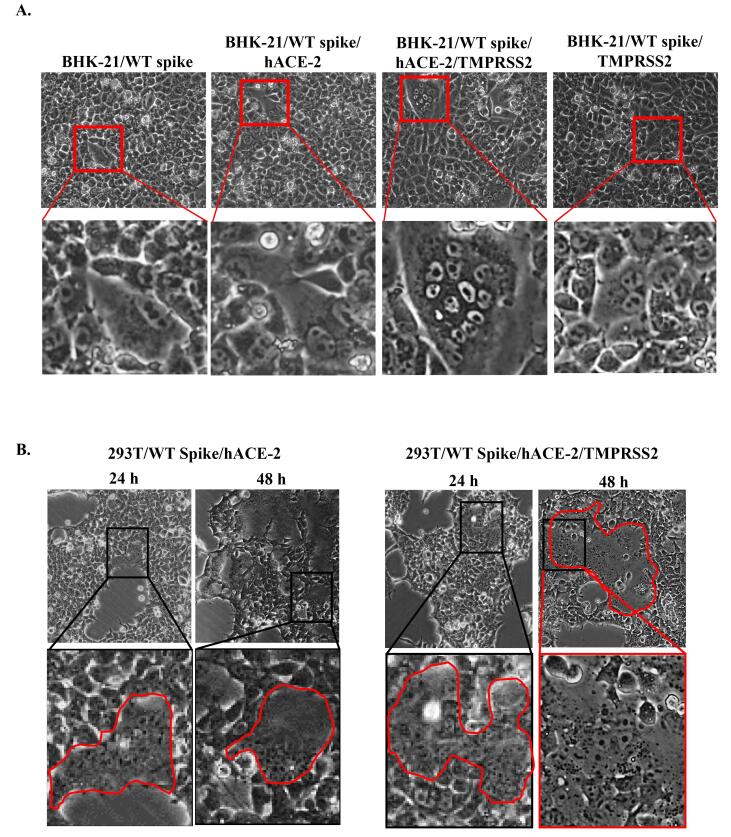
Syncytia formation in (A) BHK-21 and (B) 293T cells co-transfected with spike/hACE2 or spike/hACE2/TMPRSS2 encoding plasmids. Syncytia were observed at 24 h and 48 h post-transfection. The red line indicates syncytia

 The inhibition of intercellular transmission can be illustrated by the syncytia assay, where in this assay, we induced ectopic expression of spike, hACE2, and TMPRSS2 in BHK-21 and 293T cells. We transfected plasmids encoding spike, hACE2, and TMPRSS2 to observe protein interaction to induce syncytia. In BHK-21 cells, we observed syncytia as enlarged multinucleated cells after a 24 h incubation, especially in cells transfected with spike, hACE2, and TMPRSS2 (Figure 2A). While in 293T cells, syncytia were observed after 24 h incubation in spike/hACE2 and spike/hACE2/TMPRSS2 transfected cells and enlarged after 48 h incubation (Figure 2B). Our findings demonstrated that co-transfection of spike and hACE2 could induce syncytia formation in 293T cells while adding TMPRSS2 encoding plasmid to that co-transfection condition could enhance syncytia formation (Figure 2). Thus, we used spike/hACE2/TMPRSS2 co-expression system or spike/hACE2/lifeact-GFP for the syncytia assay without or with lifeact-GFP as a biosensor.

###  Inhibitory effect of HSD and HST in SARS-CoV-2 syncytia formation

 We observed syncytia formation following HSD or HST treatment in 293T cells transfected with plasmids for spike/hACE2/TMPRSS2 co-expression. HSD/HST 10 and 100 µM concentrations were used based on our previous results of MTT cell viability assay in 293T cells, where 100 µM of HSD/HST still maintained 293T cell viability higher than 75% with a cell seeding density 70 000 cells/ml.^21^ On the other hand, with a higher cell seeding density (140,000 cells/mL) on syncytia assay, the HSD/HST concentrations showed less or no effect on cell viability. In addition, DMSO 0.1% and DMSO 1% were used as co-solvent controls for HSD/HST 10 µM and 100 µM, respectively.

**Figure 3:**
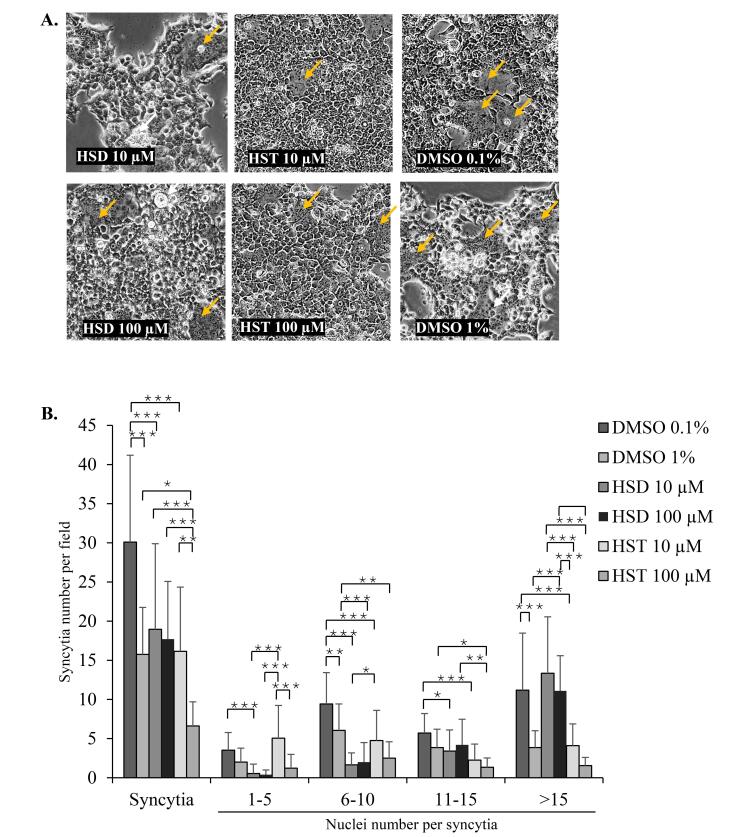
The effect of HSD and HST on syncytia formation. (A) Microscopic images of syncytia and (B) Number of syncytia and respective nuclei on 293T cells previously treated with HSD/HST10 and 100 µM or DMSO 0.1%, and 1% in two replicates. Data was analyzed from 10 microscope fields for each replicate. The red arrow indicates multinucleated syncytia. *P < 0.05, **P< 0.01, ***P < 0.001. Scale bar = 275 µm

 HSD 10 µM significantly reduced syncytia formation per microscope field to 18.95 compared to 30.10 in the DMSO 0.1% control (*P* = 0.001). At a higher concentration, HSD 100 µM did not show a significant difference in syncytia formation per field compared to the DMSO 1% control. There were also no significant differences between the two HSD concentrations, 10 and 100 µM. There was a negatively skewed distribution of the nuclei number of syncytia, where in 24 hours post-transfection, most of the SARS-CoV-2 spike-mediated syncytia had more than 15 nuclei. At 10 µM, HSD significantly reduced the number of smaller syncytia with 1–5, 6–10, and 11–15 nuclei to 0.55, 1.65, and 3.40, respectively, compared to 3.52 (*P* = 0.001), 9.43 (*P* = 0.000), and 5.71 (P = 0.045) in the DMSO 0.1% control. At 100 µM, HSD significantly reduced the formation of syncytia with 6–10 nuclei to 2.00 compared to 6.05 in the DMSO 1% control (*P* = 0.001). However, both concentrations showed a higher number of larger syncytia ( > 15 nuclei) than DMSO controls (Figure 3A and 3B).

 In cells treated with HST, both 10 and 100 µM HST reduced syncytia number to 16.15 and 6.61 in comparison to 30.10 (*P* = 0.000) and 13.75 (*P* = 0.016) in the DMSO 0.1% and 1% controls, respectively. The syncytia number per field showed lower syncytia events following the increase of HST concentration from 10 to 100 µM (*P* = 0.01). Moreover, based on the nuclei number within syncytia, HST 10 and 100 µM reduced the nuclei number of groups 6–10 nuclei/syncytium with the value of 4.75 (compared to 9.43 in DMSO 0.1%, *P* = 0.000) and 2.50 (compared to 6.05 in DMSO 1%, *P* = 0.008) and 11–15 nuclei/syncytium with the value of 2.25 (compared to 5.71 in DMSO 0.1%, *P* = 0.000) and 1.33 (compared to 3.85 in DMSO 1%, *P* = 0.029). While for > 15 nuclei/syncytium, HST 10 µM showed a lower syncytia number with the value of 4.10 compared to 11.19 in DMSO 0.1% (*P* = 0.000), and HST 100 µM did not cause a significant difference in comparison to its DMSO 1% control (Figure 3A and 3B).

**Figure 4:**
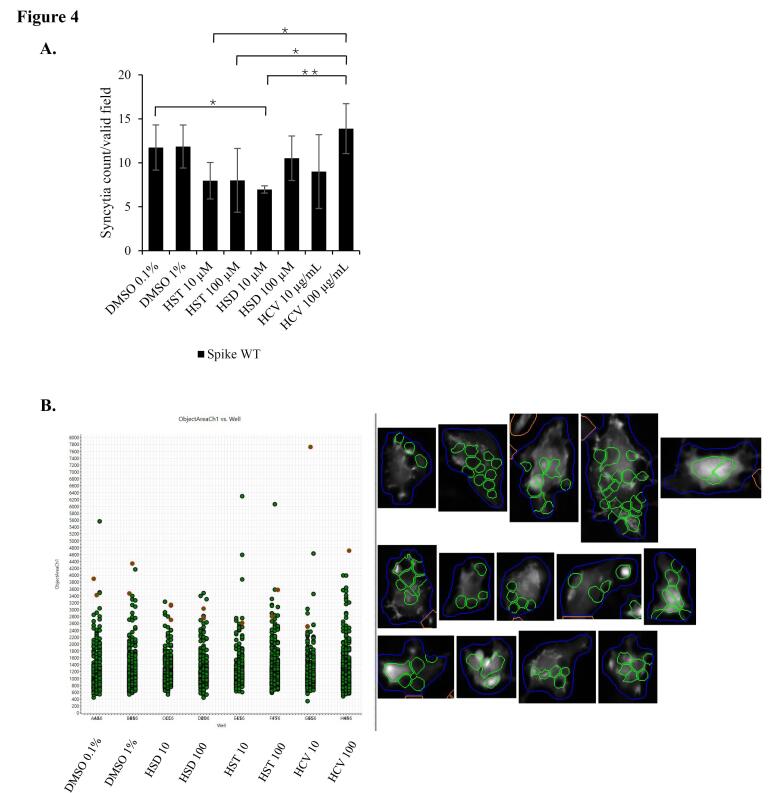
The effect of HSD, HST, and HCV on syncytia formation induced by SARS-CoV-2 spike WT. Syncytia assay in 293T cells co-transfected with SARS-CoV-2 spike WT/hACE2/lifeact-GFP (A) Syncytia number per valid field of each well in 96 well plate (Mean + SD), (B) Each spot represents the area of one single syncytium. Syncytia were observed and quantified using an HCS instrument with GFP and DAPI as fluorescence markers (n = 9 fields per well, in triplicate. Bars represent the standard deviation). *P < 0.05, **P < 0.01

**Figure 5:**
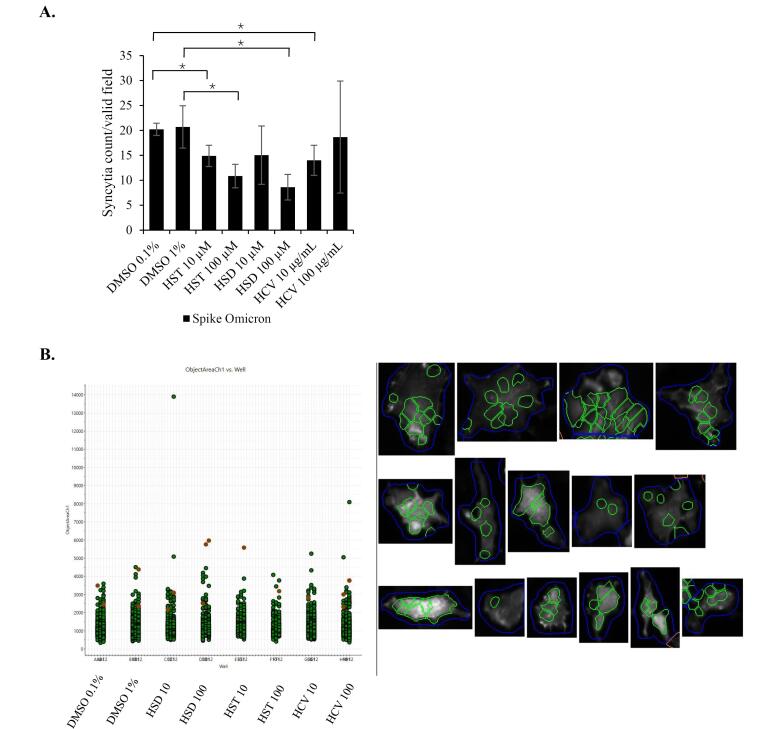
The effect of HSD, HST, and HCV on syncytia formation induced by SARS-CoV-2 spike omicron. Syncytia assay in 293T cells co-transfected with SARS-CoV-2 spike Omicron/hACE2/lifeact-GFP; (A) Syncytia number per valid field of each well in 96 well plate (Mean + SD), (B) Each spot represents the area of one single syncytium. Syncytia were observed and quantified using an HCS instrument with GFP and DAPI as fluorescence markers (n = 9 fields per well, in triplicate. Bars represent the standard deviation). *P < 0.05

 To confirm the HSD effect on the inhibition of syncytia formation, we used lifeact-GFP as a biosensor to label recombinant cells and to facilitate automated syncytia observation using the HCS instrument. In the syncytia assay, we over-expressed the SARS-CoV-2 WT spike or Omicron spike to identify the effects of HSD, HST, and HCV on different virus variants. As a result, HSD 10 μM treatment appeared to significantly decrease syncytia formation in cells transfected with WT spike, hACE-2, and lifeact-GFP compared to control cells with the value of syncytia number of 6.96 in comparison to 11.74 (*P* < 0.05) (Figure 4). Meanwhile, in cells transfected with Omicron spike, hACE2, and lifeact-GFP, treatment of HSD 100 μM, HST 10 and 100 μM, and HCV 10 μg/mL, significantly reduced the number of syncytia formed when compared to control cells with the value of 8.61, 14.89, 10.85, and 14.00 compared to 20.22 (*P* < 0.05) (Figure 5).

 These findings suggest that HSD and HST inhibit syncytia formation. HSD 10 µM as well as HST 10 and 100 µM resulted in a significantly lower incidence of overall syncytia formation. Treatment with HSD 10 µM reduced syncytia formation by 37.03% (18.95 in comparison with 30.10 syncytia number in DMSO 0.1% control). Meanwhile, HST treatment at 10 and 100 µM concentrations showed a significant decrease in syncytia formation by 46.34% (16.15 compared to 30.10 in DMSO 0.1% control) and 58.03% (6.61 compared to 15.75 in DMSO 1% control), respectively.

## Discussion

 Syncytia formation is typically facilitated by viral fusion protein, which requires host cell receptors and host proteases to induce membrane fusion. When HSD and HST bind to ACE2/TMPRSS2, syncytia formation would likely be reduced, as the viral spike protein would be less able to fuse with adjacent cells. Cheng et al^22^ reported that HST exhibits a higher binding affinity to ACE2 and TMPRSS2 compared to HSD which indicates the higher potential of HST in inhibiting syncytia formation. Our results in syncytia assay induced by spike/hACE2/TMPRSS2 showed that HSD significantly reduced the number of syncytia with 1–15 nuclei, meanwhile, HST showed significantly less formation of syncytia containing 6–15, moreover, more than 15 nuclei. In addition, the total syncytia number was lower in 100 μM HST-treated cells (Figure 3B). However, syncytia with more than 15 nuclei still be found after treatment, even though the syncytia formation had a negatively skewed distribution in nuclei number. In our syncytia assay, we treated the cells with HSD/HST about 6 h after transfection. However, during this period, we cannot prevent some cell-cell interactions in cells that might already express the ectopic proteins. It might allow the formation of larger syncytia formation. Other experiment settings, such as treating the cells at the beginning of transfection or using a mixture of two cell populations expressing receptor and spike separately^4^ may be performed to confirm the results. This aligns with the findings of Zhang et al^23^ who observed spike-mediated syncytia containing up to 60 nuclei at 12 hours post-transfection.

 We utilized DMSO as the control in this research to ensure that the remaining DMSO content in the final solution did not influence the results. DMSO was chosen due to the enhanced solubility of these compounds within it. HSD has limited solubility in other solvents like ethanol and methanol, approximately 1 mg/mL.^24^ In contrast, HSD is soluble in DMSO at about 30 mg/mL.^24^ HST also demonstrates high solubility in DMSO, reaching up to 60 mg/mL.^25^ At a higher concentration, DMSO is known to have a toxic effect on cells, including human cell lines. Referring to Zhao et al^26^ the minimum cytotoxic concentration of DMSO ranged from 0.1% to 1.6%. Moreover, the tolerability of DMSO cytotoxicity is dependent on cell types. Research conducted by Jamalzadeh et al^27^ the HUVEC normal human cell line showed a reduction in cell viability with DMSO solvent starting at 1% concentration after 24 h of treatment. However, we found a similar syncytia number between DMSO 0.1 and 1% in our assay with the HCS instrument at 16–18 hours of incubation. Moreover, our previous research has proven that HSD (1, 10, 100 μM), HST (1, 10, 100 μM), and HCV (1, 10, 100 μg/mL) dissolved in DMSO did not show a significant decrease in cell viability.^21^

 On the other hand, based on syncytia assay induced by spike/hACE2 analyzed by HCS, the syncytia number formed after HSD/HST treatment was not significantly different (Figure 4B and 5B). Recent laboratory-based studies have provided confirmatory evidence supporting the mechanism by which HSD and HST may inhibit syncytia formation in SARS-CoV-2 infection. In silico analysis by Cheng et al^22^ demonstrated that both HSD and HST can bind to TMPRSS2, with binding energies of HSD and HST at −7.2 kcal/mol and −30.56 kcal/mol. These values suggest that HST has a stronger binding affinity to TMPRSS2 compared to HSD, even though both compounds did not interact with predictive active site residues of TMPRSS2 as listed in a study conducted by Rahman et al.^28^ It possibly competes with or interferes with the viral spike protein’s ability to bind to ACE2. HST has been reported to disrupt the interaction of ACE2 with the RBD of the spike glycoprotein at a higher binding affinity than chloroquine.^12^ Moreover, syncytia formation follows an exponential rate, particularly in the presence of TMPRSS2.^5^ FRET assay has revealed that HSD and HST slightly decrease the enzymatic activity of TMPRSS2, while HST, but not HSD, decreased the interaction of ACE2 and the spike protein in vitro.^4^ TMPRSS2 facilitates the cleavage of the spike protein, a step necessary for subsequent cell-cell fusion leading to syncytia formation.^6^ Thus, it may explain the higher inhibitory effect of HST on syncytia assay compared to HSD (Figure 3B) might rely on TMPRSS2 binding potential, than hACE2 binding potential.

 Another study showed that HST has demonstrated inhibitory effects against SARS-CoV-2 3CLpro, with an IC50 value of 8.3 µM in a cell-based cleavage assay.^12^ Recent study shows HSD inhibits the cleavage activity of the Mpro in a dose-dependent manner in cell-free and cell-based assays, with an IC50 of 8.3 μM.^29^ However, specific studies on IC50 values for HSD against SARS-CoV-2 are not detailed in the provided sources. Other studies suggest that flavonoids, including HSD, can modulate intracellular calcium homeostasis, which is crucial for cell fusion and viral propagation.^30^ By disrupting calcium signaling pathways, the compound may inhibit the activation of host proteases such as TMPRSS2, further preventing syncytia formation. HST might be preferred in terms of rapid action and potency, while HSD could offer sustained therapeutic effects once converted to HST. Further head-to-head studies are needed to directly compare the IC50 values and determine their respective therapeutic windows in clinical settings.

 The potential of HSD as an antiviral is also strengthened by its ability to prevent inflammation. In vitro and in vivo studies showed that HSD reduces levels of nuclear factor (NF)-κB, inducible nitric oxide synthase (iNOS), cyclooxygenase (COX)-2, and markers of chronic inflammation.^31^ Research using animal models and clinical trials also proved that HSD has an anti-inflammatory effect through several signaling pathways, mainly NF-κB.^32^ A meta-analysis of experimental studies in healthy populations showed that HSD supplementation significantly reduced C-reactive protein (CRP), interleukin (IL)-6, IL-4, and malondialdehyde (MDA) levels in a dose-independent manner.^33^ Inflammation management is a concern in COVID-19 therapy, especially in the event of a cytokine storm, which causes an increase in the severity of the disease and the number of deaths.^34^ Therefore, compounds that can inhibit inflammation and other mechanisms, such as HSD, are needed to slow the progression of COVID-19.

 The HSD compound has been proven to maintain high viability in normal cells. In our previous study, at 1–100 µM concentrations, HSD resulted in greater than 75% viability in the 293T cells used as the cell model.^21^ In the African green monkey kidney epithelial cell line Vero E6, HSD is reported to have an inhibitory concentration 50% (IC50) of 3157 µM.^35^ HSD at a 5–50 µM concentration does not significantly affect the proliferation of WPMY-1, a human prostate stromal myofibroblast cell line.^36^ The acute toxicity test in Sprague-Dawley rats shows that 5000 mg/kg HSD caused 10% mortality. In comparison, HSD in doses of 250 and 500 mg/kg in the sub-chronic toxicity test did not cause physiological abnormalities.^31^ When HSD was administered at 1000 mg/kg, Li et al^37^ experienced significant changes in new physiological conditions, reported a median lethal dose (LD50) of HSD is 4,837.5 mg/kg and a low observed adverse effect level (LOAEL) of HSD is 1000 mg/kg.^37^ Data obtained in vitro and in vivo show a good safety profile, so HSD is suitable as a candidate for the SARS-CoV-2 antiviral.

 HSD is available in abundant quantities in orange peel,^9^ thus supporting its development as a therapeutic agent. HSD and its hydrolysis product, HST, can be a preventive or curative antiviral, especially for SARS-CoV-2. Although HSD has shown a favorable safety profile, its therapeutic effectiveness may be limited by its relatively low bioavailability. Studies have indicated that the peak plasma concentration of HST, the metabolite of HSD, after ingesting 500 mL of orange juice, reaches approximately 2.2 μmol/L, with significant variability among individuals.^38^ To achieve effective therapeutic concentrations, individuals would likely need to consume large quantities of citrus fruits, which may not be practical. To overcome this limitation, innovative formulation strategies have been proposed to enhance HSD delivery. Researchers have explored the use of nanoparticles and self-micro emulsifying drug delivery systems (SMEDDS) as promising approaches.^31^ Additionally, co-administration with bioavailability enhancers such as piperine (from black pepper) or quercetin has been suggested, as these compounds can inhibit metabolic enzymes, thereby improving HSD bioavailability.^31^

 By HCS analysis, we observed different inhibition effects of HSD and HST at different concentrations in syncytia formation mediated by different spike variants. Only HSD 10 μM showed significant syncytia inhibition with control cells in the WT spike syncytia assay. On the other hand, in the Omicron spike syncytia assay, HSD 100 μM showed a syncytia inhibitory effect compared to the control cells. The same effect was observed on HST 10 μM, HST 100 μM, and HCV 10 μg/mL. HSD is present at the concentration of 4.34% w/w HCV,^21^ orange peel extract obtained from hydrodynamic cavitation.^14^ In the syncytia assay in spike/hACE2/lifeact-GFP-transfected cells, the inhibitory effects of HSD 10 and HST 10 μM; and HSD 100 and HST 100 μM on both syncytia induced by the WT spike and Omicron spike did not show significant differences. These results indicate that HSD and HST have a similar inhibitory effect on syncytia formation induced by different SARS-CoV-2 variants. Even though HCS analysis was beneficial for automated syncytia counting, however, it has limitations regarding instrument setting to determine the target object by setting up the acquisition parameters especially to recognize cell boundaries to decide a valid syncytium.^20^

 Omicron and its variants have several unique mutations in the RBD region. The RBD mutations might control the functionality of that specific RBD region.^39^ The strength of the binding affinity of the RBD region of the Omicron variant to the receptor ACE2 is 1.5–2.8 times higher than that of the wild-type strain.^22^ The mechanistic differences underlying the differential inhibitory effects of HSD and HST against the wild-type and Omicron spike proteins are likely multifactorial. These may include changes in the spike protein’s structure, conformation, and binding affinity due to the specific mutations present in the Omicron variant. Additionally, immune evasion and alterations in viral entry mechanisms could further influence how effective these inhibitors are against the Omicron spike compared to the wild-type.^40^ To fully understand these differences, further detailed structural and biochemical studies would be required to examine how these compounds interact with the mutated spike proteins.

 The present study is limited to cell-based in vitro assays. Our previous research showed that HSD and HST could be a viable treatment for SARS-CoV-2 infection due to their strong binding to spike, ACE2, or TMPRSS2 in silico.^10,11^ The current study, along with the cellular entry study using the pseudovirus model,^21^ finally proves the potential of HSD, as well as its aglycon HST, to inhibit syncytia formation mediated by the proteins as mentioned above. Nevertheless, an in vivo study should be carried out, as the efficacy of HSD/HST in limiting COVID-19 progression would also be determined by its pharmacokinetic and pharmacodynamic profile.

## Conclusion

 Syncytia formation assay implies the potential inhibitory effects of HSD and HST on SARS-CoV-2 cell-to-cell transmission by inhibiting the fusion mechanism. Considering that this method is not directly related to the primary infection of SARS-CoV-2, the potential for inhibition of syncytia is correlated with efforts to improve the prognosis of COVID-19 patients. Thus, these results complement previous research results on the potential of HSD/HST in inhibiting SARS-CoV-2 infection through inhibition of spike/hACE2/TMPRSS2 interactions and open up opportunities for the application of HSD/HST as a supplement in preventing and improving the prognosis due to SARS-CoV-2 infection. However, due to limitations of this study that emphasize in vitro cellular study, further studies are needed to confirm the antiviral activity of HSD and HSD in in vivo models. Furthermore, research to analyze the potential combination of HSD/HST with other flavonoids or with antiviral drugs is also needed to further open up opportunities for the application of HSD/HST as an antiviral supplement.

## Competing Interests

 Authors declare no conflict of interest in this experiment and publication.

## Ethical Approval

 Not applicable.
